# Pancreatic Myeloid Sarcoma

**DOI:** 10.7759/cureus.8462

**Published:** 2020-06-05

**Authors:** Ammar Al-Obaidi, Nathaniel A Parker, Yasmine Hussein Agha, Hamzah Alqam, Seth Page

**Affiliations:** 1 Internal Medicine, University of Kansas School of Medicine, Wichita, USA; 2 Internal Medicine, Ascension Via Christi St. Francis, Wichita, USA

**Keywords:** myeloid sarcoma, extramedullary leukemia, acute myeloid leukemia, granulocytic sarcoma, pancreatic sarcoma, chemotherapy, allogenic bone marrow transplant

## Abstract

Myeloid sarcoma is an isolated extramedullary tumor mass consisting of immature myeloid cells. It is characterized by highly variable outcomes and usually disrupts the normal architecture of the normal tissue in which it originates. It may occur de novo or be associated with other hematological malignancies. Clinical presentation of myeloid sarcomas can be highly variable based on the tumor site, size, and extent of tissue involvement. The diagnosis of myeloid sarcoma is challenging and requires a high index of suspicion. Tissue sampling followed by the use of auxiliary studies is essential for diagnosis. Moreover, bone marrow sampling is necessary to exclude morrow involvement. Currently, the recommended therapeutic regimens for myeloid sarcoma are similar to those for acute myeloid leukemia. Much work remains to be accomplished as myeloid sarcomas, if initially missed or misdiagnosed, have poor overall survival rates. Furthermore, prognostic factors for this malignancy remain poorly understood.

## Introduction

Myeloid sarcoma (MS), also called granulocytic sarcoma or chloroma, is an extramedullary solid tumor that arises from myeloid precursor cells [1]. Its pathogenesis is highly variable; it can develop as an isolated mass or in relation to other hematological malignancies such as myeloproliferative disorder, myelodysplastic syndrome (MDS), and acute myeloid leukemia (AML) [2]. MS can be the initial manifestation of extramedullary blood cancer, can occur concurrently with it, or can represent a relapsing disease in a patient in remission [3]. Isolated MS, in particular, is exceedingly rare as it occurs in 2 per 1,000,000 individuals [4]. Cases in the literature have described the lymph nodes, bones, skin, lungs, external genitalia, bowels, and brain as sites of incidence. We present an unusual case of isolated pancreatic MS several years post-stem cell transplant in a nonleukemic patient.

## Case presentation

A 57-year-old female presented to the emergency department with the chief complaints of progressive generalized weakness, epigastric abdominal pain radiating to her back, and a progressively worsening yellow tint to the skin and eyes. The onset of her complaints reportedly occurred seven days prior to her initial presentation. Eight years prior she was diagnosed with MDS, for which she underwent successful allogenic bone marrow transplantation that was complicated by subsequent chronic graft versus host disease. Complete remission of her MDS and graft versus host disease were achieved by allogenic bone marrow transplant and chronic ruxolitinib therapy, respectively. Other noteworthy information included a chronic history of tobacco smoking, alcohol dependence, non-intravenous use of methamphetamines, cholecystectomy, and pancreatic cancer in her father.

**Figure 1:**
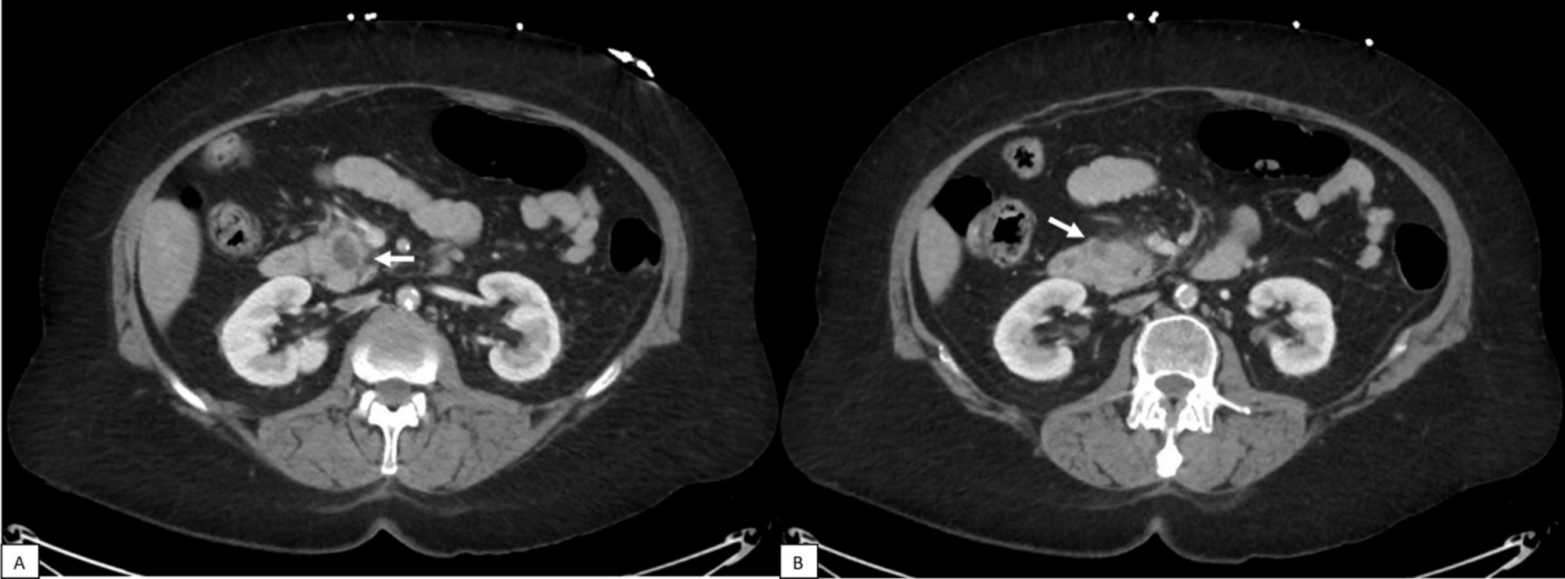
Abdominopelvic imaging demonstrating a new pancreatic mass. (A) CT scans of the abdomen and pelvis with contrast shows a new ellipsoid area of hypo-enhancement within the pancreatic head near the region of the ampulla that measures 2.2 x 1.5 cm. (B) A separate area of hypo-enhancement seen more laterally and inferiorly measures 1 cm. The pancreatic duct is not distended.

Physical examination was remarkable only for icteric sclera and mild, generalized abdominal tenderness. Vital sign measurements were unremarkable. Laboratory testing showed primarily biochemical evidence of cholestatic and hepatocellular liver injury, as well as pancreatic islet cell dysfunction (Table 1). Abdominopelvic computed tomography (CT) scans obtained in the emergency department showed a hypo-enhancing lesion within the pancreatic head causing secondary intra- and extrahepatic biliary ductal dilatation (Figure 1). She was admitted for obstructive jaundice, as well as further evaluation and management of her problems.

**Table 1:** Serum biochemical analysis suggests pancreatic dysfunction, acute liver injury, and biliary abnormalities. The ratio of alanine aminotransferase to alkaline phosphatase elevation suggests a cholestatic pattern of liver injury.

Labs	Result	Reference range
White blood cells	10.9	4.8–10.8 x 10^3^/uL
Hemoglobin	14.6	12–16 gm/dL
Platelets	220	150–400 x 10^3^/uL
Bands	1	0–8%
Manual differential	Normal	Normal
Glucose, non-fasting	389	71–114 mg/dL
Hemoglobin A1c	13	4.1–5.6%
Aspartate aminotransferase	561	15–41 U/L
Alanine aminotransferase	445	14–54 U/L
International normalized ratio	1.1	0.9–1.2
Alkaline phosphatase	1,208	26–104 U/L
Total bilirubin	20.1	0.2–1.2 mg/dL
Direct bilirubin	8.8	0–1 mg/dL
Indirect bilirubin	11.3	0–0.2 mg/dL
Lipase	38	8–38 U/L
Triglyceride	564	0–150 mg/dL

**Figure 2:**
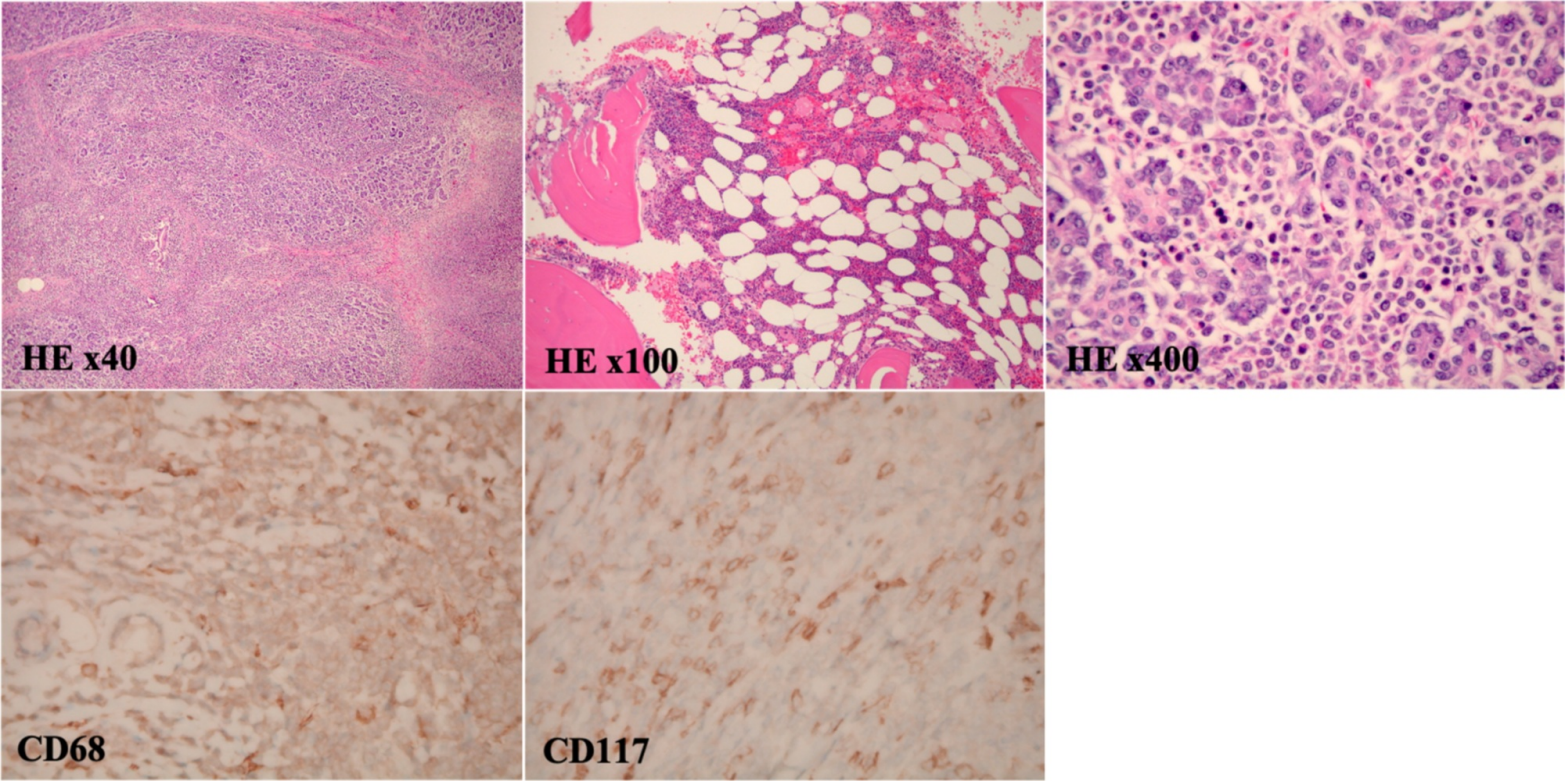
Pathology provided by Whipple resection shows a pancreatic myeloid sarcoma. H&E staining of pancreatic tissue specimens shows an infiltrate comprised of diffuse sheets of atypical cells with scant cytoplasm, irregular nuclei, and prominent nucleoli. Myeloid blast cells infiltrate between and through benign pancreatic ducts and acini (H&E x40, x100, and x400). No lymph node involvement is present. The lesional cells show immunoreactivity with cytoplasmic CD68 and dimly with CD117 on immunohistochemical staining. Thus, aberrant B-cell and T-cell expressions are observed, which further supports the diagnosis of MS. H&E, hematoxylin and eosin

She underwent a CT scan of the chest to evaluate for metastatic disease, which was unremarkable for any lesions or lymphadenopathy. Pancreaticoduodenectomy (Whipple procedure) was performed for symptom management, mass resection, and diagnosis. Grossly, a 3.5 x 3.0 x 2.5 cm ill-defined mass was observed to be intruding on the ampulla and common bile duct. On light microscopy, diffuse sheets of atypical cells with scant cytoplasm, irregular nuclei, and prominent nucleoli were evident. These cells infiltrated between and through benign pancreatic ducts and acini. Immunohistochemical (IHC) staining on the resected pancreatic tissue revealed the tumor cells were strongly positive with CD43 and CD68 (Figure 2) but only minimally immunoreactive with CD45 and CD117. The tumors cells were negative for CD3, CD20, and CD34. Histochemistry together with this IHC profile supported the diagnosis of pancreatic MS rather than epithelial malignancy. Given the patient’s new diagnosis of an extramedullary myeloid tumor, blood and bone marrow involvement was evaluated by serum analysis and bone marrow aspiration and biopsy. Peripheral blood smear as well as bone marrow aspiration and biopsy were negative for an increase in blast cells. Bone marrow aspirate was subjected to 17-gene next-generation sequencing assays for AML. No mutations were identified (Table 2).

**Table 2:** PCR-based next generation sequencing assays detected no genetic alterations. Negative genes represent clinically relevant mutations associated with acute myeloid leukemia. Mutational hotspots and surrounding exonic regions were interrogated for DNA level point mutations and indels (fusions were not assayed). No genetic alterations were identified. PCR, polymerase chain reaction

Pertinent negative results		
ASXL1	BCOR	DNMT3A
EZH2	FLT3	IDH1
IDH2	KIT	KRAS
NPM1	NRAS	PHF6
PTPN11	RUNX1	TET2
WT1		

The patient tolerated her hospital admission well and was dismissed with instructions to follow up with her local oncologist. She remains alive and positron emission tomography (PET)/CT scans will be performed for staging and help guide therapy. Induction chemotherapy is planned following improvement in the coronavirus (COVID-19) pandemic.

## Discussion

MS is an extramedullary blast cell proliferation of one or more of the myeloid lineages, resulting in disruption of the normal architecture of the tissue in which it originates [5]. While MS is listed in the World Health Organization (WHO) as a subtype of AML, in reality it is a distinct entity that warrants further pathological and genetic analysis in an attempt to define its own WHO subclassification [6]. Primarily, MS precedes AML, or both pathologies are discovered simultaneously. MS may also present de novo, as a relapse of AML, or as a progression of a prior MDS, chronic myeloid leukemia, and other myeloproliferative neoplasms [3,5,6]. Isolated MS is extremely rare, with an overall incidence of less than 1% as compared with 2.5-9% in patients with AML [7]. Unlike our patient who had allogenic bone marrow transplant for MDS, the reported incidence of MS following allogenic bone marrow transplant for AML is 5-12% with or without bone marrow involvement [8,9].

Given the low incidence of MS, epidemiologic data remain sparse. Based on the limited number of large retrospective studies that have reviewed U.S. cases of MS, Caucasian males at an average age of 60 years are at an increased risk of developing this rare condition. Connective tissue, breasts, integumentary, and digestive system are the most common primary sites [7]. Following diagnosis, the prognosis is often difficult to determine. In one large retrospective study, the median overall survival was found to be approximately 13 months [7]. Ultimately, positive predictors for improved survival are the absence of AML, and MS arising from genitourinary, gastrointestinal, and ocular tissue [10].

In the absence of bone marrow involvement, the diagnosis of isolated MS is challenging. It is frequently misidentified as one of the more common hematological neoplasms, especially in the setting of conventional light microscopy. Based on the advances in imaging and diagnostic methods, the rate of misdiagnosis has decreased from 75% to 25-50% [4,11].

To our knowledge, we report the eighth case of pancreatic MS in a patient without a coexisting or primary hematological malignancy that has been described in the literature [12-14].

The presentation of MS can be highly variable. Patients can be asymptomatic or complain of symptoms secondary to tumor mass effect. Abdominal imaging can be useful but ultimately is not diagnostic due to its inability to distinguish isolated MS from other pathologies, such as pancreatitis. No tumor markers have been established for this entity, making the diagnosis even more challenging. The gold standards for diagnosing isolated MS are tissue biopsy, histopathological investigation, and IHC staining [1]. Histologically, MS consists of diffusely infiltrative myeloblasts with granulocytic or monocytic differentiation. These cells are typically large, have abundant cytoplasm, have large nuclei, and tend to partially or totally efface the tissue architecture. The presence of eosinophilic metamyelocytes may hint more toward granulocytic differentiation, whereas monocytic neoplasms are more difficult to recognize. In either case, cytochemistry, immunophenotyping, cytogenetics, and molecular studies are imperative [5,15,16]. The most frequently expressed antigens by MS include CD11c, CD13, CD33, CD34, CD43, CD45, CD56, CD68, CD117, lysozyme, and myeloperoxidase. Aberrant expression of B-cell or T-cell markers may also be observed [17-19]. Once diagnosed, it becomes fundamental to determine whether MS is occurring de novo or coinciding with a myeloid neoplasm. Thus, bone marrow biopsy remains paramount due to highly variable outcomes [12,13].

Complex cytogenetics occur in about 17% of MS in nonleukemic patients compared with 39% of patients with MS arising in the setting of AML. Common genetic abnormalities include t(8,21) (q22;q22), inv(16), 11q23, t(9;11), t(8;17), t(8;16), t(8;17), t(1;11), monosomy 7, deletion of chromosomes 5q, 16q, and 20q, and trisomy 4, 8, and 11 [18]. Involvement of some particular extramedullary sites is associated with certain abnormalities, such as MS of the abdomen and inv(16) [15-19].

Initiating treatment in a timely manner is of utmost importance in isolated MS to prevent or reduce the risk of progression to leukemia. In most isolated pancreatic cases, a final diagnosis is obtained following surgical resection and histopathology interpretation of the lesion, such as in our patient. However, recently endoscopic ultrasound with fine needle aspiration has determined to be useful or pancreatic mass sampling [12]. Using this modality, when applicable, would help establish a diagnosis rapidly. Also, it would avoid unnecessary invasive surgical interventions and complications and subsequently improve prognosis by initiating chemotherapy earlier.

Based on previous institutional studies, the median time to develop AML from extramedullary MS ranged from 5 to 12 months [11]. Thus, the current National Comprehensive Cancer Network guidelines recommend initially treating extramedullary MS with induction chemotherapy similar to that for patients with overt AML. This also applies to MS cases with concurrent bone marrow involvement. Despite the fact that isolated pancreatic MS can be treated with radiotherapy or surgical resection, AML-type remission induction chemotherapy is mandatory after resection of the isolated MS lesion. Otherwise, MS would recur [12,15-19]. In addition, time to progression to acute leukemia has been shown to be longer in those treated with systemic chemotherapy as opposed to local radiotherapy or surgery [11]. Several retrospective studies have demonstrated the efficacy of allogenic stem cell transplant in isolated MS cases, especially after inducing remission. However, there are no controlled clinical trials to evaluate the role of this mode of therapy in this setting [1,4].

## Conclusions

Isolated MS is a rare condition and may occur at any site, leading to widely variable clinical presentations based on the tumor site, size, and extent of tissue involvement. It should, therefore, be considered as a differential diagnosis of any atypical cellular infiltrate. Its diagnosis is challenging and requires a high index of suspicion. The use of auxiliary studies (cytochemistry, cytogenetics, and molecular analysis) is essential for diagnosis. Additionally, bone marrow sampling is necessary to exclude morrow involvement. As for management, AML-type chemotherapy for remission induction should be used for MS (whether isolated or co-occurring with bone marrow involvement). Moreover, relapsing MS should be treated similar to relapsed AML. Post-remission therapy includes radiotherapy and chemotherapy depending on the extent of involvement, performance status, and risk profile. Randomized controlled trials are needed to elucidate the role of allogenic stem cell transplant and to further refine management protocols. Recent advances in genetic profiling may enable the development of novel targeted therapies. The prognostic factors and outcomes of extramedullary MS, apart from relapse and AML transformation, remain poorly understood.

## References

[1] Pileri SA, Ascani S, Cox MC (2007). Myeloid sarcoma: clinico-pathologic, phenotypic and cytogenetic analysis of 92 adult patients. Leukemia.

[2] Magdy M, Abdel Karim N, Eldessouki I, Gaber O, Rahouma M, Ghareeb M (2019). Myeloid sarcoma. Oncol Res Treat.

[3] Yilmaz AF, Saydam G, Sahin F, Baran Y (2013). Granulocytic sarcoma: a systematic review. Am J Blood Res.

[4] Antic D, Elezovic I, Milic N (2013). Is there a "gold" standard treatment for patients with isolated myeloid sarcoma?. Biomed Pharmacother.

[5] Shahin OA, Ravandi F (2020). Myeloid sarcoma. Curr Opin Hematol.

[6] Arber DA, Orazi A, Hasserjian R (2016). The 2016 revision to the World Health Organization classification of myeloid neoplasms and acute leukemia. Blood.

[7] Goyal G, Bartley AC, Patnaik MM, Litzow MR, Al-Kali A, Go RS (2017). Clinical features and outcomes of extramedullary myeloid sarcoma in the United States: analysis using a national data set. Blood Cancer J.

[8] Lee KH, Lee JH, Choi SJ (2003). Bone marrow vs extramedullary relapse of acute leukemia after allogeneic hematopoietic cell transplantation: risk factors and clinical course. Bone Marrow Transplant.

[9] Shimizu H, Saitoh T, Hatsumi N (2013). Prevalence of extramedullary relapses is higher after allogeneic stem cell transplantation than after chemotherapy in adult patients with acute myeloid leukemia. Leuk Res.

[10] Movassaghian M, Brunner AM, Blonquist TM (2015). Presentation and outcomes among patients with isolated myeloid sarcoma: a Surveillance, Epidemiology, and End Results database analysis. Leuk Lymphoma.

[11] Yamauchi K, Yasuda M (2002). Comparison in treatments of nonleukemic granulocytic sarcoma: report of two cases and a review of 72 cases in the literature. Cancer.

[12] Nunes G, Pinto-Marques P, Mendonça E, Gargaté L (2019). First case report of pancreatic myeloid sarcoma diagnosed through EUS-FNA. Gastroenterol Hepatol.

[13] Moreau P, Milpied N, Thomas O (1996). [Primary granulocytic sarcoma of the pancreas: efficacy of early treatment with intensive chemotherapy]. Rev Med Interne.

[14] Tokunaga K, Yamamura A, Ueno S (2018). Isolated pancreatic myeloid sarcoma associated with t(8;21)/RUNX1-RUNX1T1 rearrangement. Intern Med.

[15] Bakst RL, Tallman MS, Douer D, Yahalom J (2011). How I treat extramedullary acute myeloid leukemia. Blood.

[16] Almond LM, Charalampakis M, Ford SJ, Gourevitch D, Desai A (2017). Myeloid sarcoma: presentation, diagnosis, and treatment. Clin Lymphoma Myeloma Leuk.

[17] Claerhout H, Van Aelst S, Melis C (2018). Clinicopathological characteristics of de novo and secondary myeloid sarcoma: a monocentric retrospective study. Eur J Haematol.

[18] Ullman DI, Dorn D, Jones JA (2019). Clinicopathological and molecular characteristics of extramedullary acute myeloid leukaemia. Histopathology.

[19] Wang HQ, Li J (2016). Clinicopathological features of myeloid sarcoma: report of 39 cases and literature review. Pathol Res Pract.

